# Author Correction: Arrhythmic sudden death survival prediction using deep learning analysis of scarring in the heart

**DOI:** 10.1038/s44161-022-00075-z

**Published:** 2022-04-28

**Authors:** Dan M. Popescu, Julie K. Shade, Changxin Lai, Konstantinos N. Aronis, David Ouyang, M. Vinayaga Moorthy, Nancy R. Cook, Daniel C. Lee, Alan Kadish, Christine M. Albert, Katherine C. Wu, Mauro Maggioni, Natalia A. Trayanova

**Affiliations:** 1 Alliance for Cardiovascular Diagnostic and Treatment Innovation (ADVANCE), Johns Hopkins University, Baltimore, MD USA; 2 Department of Biomedical Engineering, Johns Hopkins University School of Medicine, Baltimore, MD USA; 3 University of Pittsburgh Medical Center, Heart and Vascular Institute, Pittsburgh, PA USA; 4 Cedar-Sinai Medical Center, Department of Cardiology, Los Angeles, CA USA; 5 Brigham and Women’s Hospital, Harvard Medical School, Boston, MA USA; 6 Northwestern University, Feinberg School of Medicine, Chicago, IL USA; 7 Touro College and University System, Valhalla, NY USA; 8 Johns Hopkins University School of Medicine, Department of Medicine, Division of Cardiology, Baltimore, MD USA; 9 Department of Applied Mathematics and Statistics, Johns Hopkins University, Baltimore, MD USA

**Keywords:** Ventricular tachycardia, Disease-free survival, Medical imaging

Correction to: *Nature Cardiovascular Research* 10.1038/s44161-022-00041-9, published online 7 April 2022.

This paper was originally published under a standard Springer Nature license (© The Author(s), under exclusive licence to Springer Nature Limited). It is now available as an open-access paper under a Creative Commons Attribution 4.0 International license, © The Author(s). The error has been corrected in the online version of the article.

